# A tailored counseling and home-based rehabilitation program to increase physical activity and improve mobility among community-dwelling older people after hospitalization: protocol of a randomized controlled trial

**DOI:** 10.1186/s12891-017-1825-5

**Published:** 2017-11-21

**Authors:** K. Turunen, L. Aaltonen, J. Kumpumäki, E. Portegijs, S. Keikkala, M.-L. Kinnunen, T. Finni, S. Sipilä, R. Nikander

**Affiliations:** 1 GeroCenter Foundation for Aging Research and Development, Jyväskylä, Finland; 2 Gerontology Research Center and Unit of Health Sciences, Faculty of Sport and Health Sciences, University of Jyväskylä, Jyväskylä, Finland; 3 Health Centre Hospital, Health Centre of Jyväskylä Cooperation Area, City of Jyväskylä, Finland; 4 Neuromuscular Research Center, Faculty of Sport and Health Sciences, University of Jyväskylä, Jyväskylä, Finland

**Keywords:** Physical activity, Sedentary behavior, Rehabilitation, Clinical trial, Mobility, Aging, Musculoskeletal, Injury

## Abstract

**Background:**

Physical activity (PA) decreases during hospitalization. In particular, the amount of PA engaged in by older people who are hospitalized following musculoskeletal injury is likely to be limited for months after discharge home. Given the importance of an active lifestyle for their recovery and the prevention of future adverse outcomes, there is clearly a need for interventions to increase PA. This article describes the protocol of a randomized controlled trial set up to investigate the effects of a physical activity oriented home rehabilitation program (ProPA) on PA and the restoration of mobility in community-dwelling older people.

**Methods:**

Men and women aged 60 years or older hospitalized due to a musculoskeletal injury or disorder in the back or lower limbs are recruited. After discharge from hospital to home, participants are randomized into a six-month ProPA program or a standard care (control) group. The ProPA program consists of a motivational interview, goal attainment process, guidance for safe walking, a progressive home exercise program and physical activity counseling. In addition, frail participants who are not able to go outdoors alone receive support from volunteers.

Primary outcomes are PA measured using a 3-dimentional accelerometer, and mobility assessed by the Short Physical Performance Battery and self-reports. Secondary outcomes are life space mobility, participation restriction, fear of falling, pain, mood, and grip strength. Information on barriers to and enablers of PA participation are also collected. Data on mortality and use of health services are collected from the national register. In this 6-month intervention, all participants are assessed in their homes at baseline and after three and six months, and at 12 months after randomization they will receive a follow-up questionnaire.

**Discussion:**

This study investigates the effects of a rehabilitation program on PA and mobility among older people at risk for increased sedentary time and mobility problems. If positive effects are observed, the program can be considered for incorporation into the health care system and thereby contribute to the rehabilitation of older people who have recently been discharged from hospital.

**Trial registration:**

ISRCTN13461584. Registered 27 January 2016.

## Background

Twenty percent of people in Finland are aged 65 years or over, and many of them have been hospitalized because of an acute or chronic musculoskeletal injury or disorder. During and after hospitalization, older people often experience a substantial decline in function; this can result in loss of independence in daily life, decreased quality of life, and thus increased likelihood of readmission to hospital or even to moving from home into a long-term care facility [1–3]. For example, up to half of hip fracture patients do not achieve their pre-fracture level of mobility, or regain independence in daily life [4–6]. The regaining of mobility during and after hospitalization is crucial for enabling old people to move around inside and outside the home, maintain physical activity (PA), re-engage in social activity and preserve their autonomy. Indeed, adequate mobility is a key component of functional recovery and participation in society [7].

Whereas physical activity includes household tasks, activities of daily living, exercise and sports [8], sedentary behavior refers to any waking behavior characterized by an energy expenditure of ≤1.5 metabolic equivalents (METs) while in a sitting, reclining or lying posture [9]. Sedentary behavior is common, particularly among older people who have been hospitalized. Typically, sedentary time exceeds 20 h per day among those admitted to hospital [3] and older patients walk only an average of 700 to 1500 steps per day [10–12]. Obviously, patients who have had an orthopedic surgery are even less active during hospitalization than those who have not been treated surgically [13, 14]. Little information is available on the physical activity level of older people after discharge from hospital. As a patient’s mobility limitations persist long after discharge to home [5, 15, 16], it is likely that their amount of physical activity will also be insufficient for months. Sedentary behavior, in turn, increases the risk for future injury and subsequent hospitalization. Thus, optimizing recovery by promoting PA and improving mobility after a musculoskeletal injury or disorder in the lower limbs or back has the potential to reduce the burden on individuals and society.

Currently, little evidence exists on the best ways to promote PA and support mobility recovery after hospitalization among older people with musculoskeletal injury or disorder. Although there is no consensus on rehabilitation best practices, recent home-based rehabilitation interventions with minimal contact from a physiotherapist (4 to 5 visits) boosted by telephone calls resulted in improvement in the physical function of the lower limbs [17] self-reported mobility [18] and self-reported level of PA [19] among hip fracture patients.

The present study, investigates the effect of a six-month individually tailored and home-based counseling and rehabilitation program in comparison to standard care on PA and mobility recovery among community-dwelling men and women aged 60 years or older who have been recently discharged from hospital and are recovering from a musculoskeletal injury or lower limb or back disorder. Objective measures of PA using 3D accelerometers are utilized to assess sedentary time and the duration and intensity of PA. This will especially help detection of nuances in light activity during recovery after acute hospital stay among older people with musculoskeletal problems. This article describes the recruitment process, data collection, and intervention of this ongoing randomized controlled trial.

## Methods

### Context

In Finland, municipalities are responsible for organizing primary health care and hospital districts for specialized medical care. Municipalities can provide primary health care services alone or jointly with other municipalities. In Jyväskylä, primary health care is delivered by the health center of Jyväskylä Cooperation Region (JYTE) for a catchment area comprising three municipalities with a total population of 140,000. Primary health care includes inpatient rehabilitation and ward care provided in health center hospitals and outpatient care in clinics at the local health care centers. The health center hospitals offer curative and rehabilitative care for patients who do not need specialist medical care. For example, after orthopedic surgery (i.e. hip or other fracture, joint replacement), performed at the Central Hospital of Central Finland, patients resident in Central Finland are transferred to their local health center hospital for inpatient care and rehabilitation, typically within the first post-operative days. The inpatient rehabilitation period ranges from a few days to a few months depending on the individual’s health status and care needs. After the inpatient period, patients are discharged to home. When needed, their independent living at home is supported through home care services provided by the social services department of their municipality of residence. The participants of this study are being recruited from the health center hospitals of JYTE.

### Design

**Fig. 1:**
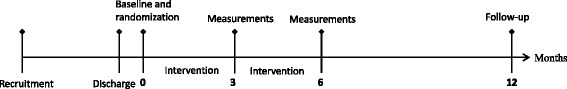
RCT schedule for randomization, measurements and follow-up

This study is a parallel group randomized controlled trial (RCT, ISRCTN13461584) with two groups: a promotion of physical activity (ProPA, intervention) group and a standard care (control) group. Figure 1 provides an overview of the study design. Recruitment started during spring 2016 and will be finished by the end of August 2017. After the baseline measurements, participants are randomized into the two study groups. The computer-generated randomization is performed by an independent statistician. Randomization by sex, age (60 to 84 years or 85 years of age and over) and baseline gait speed (< 0.4 m/s or ≥0.4 m/s) is performed in blocks of 10 participants.

All participants are measured three times at home: at baseline immediately after discharge from hospital, and at three and six months thereafter. Moreover, participants are followed up for a further six months (up to 12 months from baseline) to collect data on PA, mobility limitations, life-space mobility, fear of falling, pain and mood.

### Sample size calculation

A priori sampled size calculations are based on previously published work [20] and on our pilot study conducted among 55 community-dwelling older people who were followed up for six months after discharge from hospital. In our pilot study, using an accelerometer, daily active time measured as mean amplitude deviation (MAD) above 0.0167 g was 250 ± 103 min. It has been estimated that a 20% increase in habitual physical activity, as measured by an accelerometer, in the intervention group compared to standard care control group will be clinically meaningful [21]. For this effect size, a significance level of 0.05 for the PA outcomes and a power of 80% will be set. Thus, the required sample size is 53 participants in each group. Assuming a dropout rate of 10–15%, 60 participants per group (total 120 participants) are required.

### Participants and the recruitment process

Recruitment is implemented at two health center hospitals, Kyllö and Palokka, in the city of Jyväskylä, Finland. A research nurse reviews the medical records of all ambulatory and community-dwelling men and women aged 60 years and older who live in the city of Jyväskylä or its neighboring municipalities, and who have been admitted to either of the two hospitals owing to a lower limb or back musculoskeletal injury or disorder, including for limb or back surgery (e.g. hip fracture, joint replacement, aggravated arthritis), or a fall-induced injury. Patients who fulfill the inclusion criteria are then sent an information letter on the study from the research nurse. Those interested in participating have an opportunity to discuss their participation with the researcher before signing the informed consent and giving permission to review their medical records. Patients living in an institution or bedridden at the time of hospital admission, or who suffer from severe memory problems (Mini-Mental State Examination <20), alcoholism and unstable cardiovascular, pulmonary or progressive neurological disease are excluded from the study.

### Ethics

This study was approved by the ethics committee of the Health Care District of Central Finland on September 4th, 2014 (3 N/2014). Written information on the study was given to all participants. Participants signed an informed consent form prior to their participation. Proxy consent was not permitted.

### Measures

Baseline measurements start in the hospital ward and continue immediately after discharge to home. The 3- and 6-month assessments are performed in the participant’s home. Before the study start, examiners are given training in the testing procedures. At baseline, chronic conditions, use of prescription medication and the type of musculoskeletal condition that resulted in hospitalization (diagnosis) and its treatment (e.g. surgery type and medication) are drawn from the medical records of the Central Hospital of Central Finland and health center hospital. All the other measures and schedule are listed in Table 1.

**Table 1:** Measures included in the study at each time-point

Intsrument	Domains	Time point					
		Prior to hospital admission	Baseline	3 months	6 months	12 months	Ref.
Main outcome measures							
Physical activity	Accelerometer 6 days		x	x	x	–	
	Diary of physical activity 6 days		x	x	x	–	
	Modified Grimby scale	x	x	x	x	x	[22, 23]
	YPAS (standing/walking & lying down/sitting)	x	x	x	x	x	[45]
	Unmet physical activity		x	x	x	x	[32]
Short Physical Performance Battery (SPPB)	Balance, 4 m walking, chair rising		x	x	x	–	[25]
Mobility disability	Moving outdoors	x	x	x	x	x	[46]
	Walking 500 m	x	x	x	x	x	[46]
	Walking 2 km	x	x	x	x	x	[46]
	Stair climbing	x	x	x	x	x	[46]
	Lifting (10 kg)	x	x	x	x	x	[46]
Self-rated mobility			x	x	x	x	
Secondary outcomes							
Life-Space Mobility (LSA)	Within home	x	x (modified)	x	x	x	[26]
	Outdoor	x	x (modified)	x	x	x	[26]
	Neighborhood	x	x (modified)	x	x	x	[26]
	Town	x	x (modified)	x	x	x	[26]
	Unlimited	x	x (modified)	x	x	x	[26]
Impact on Autonomy & Participation (IPA), 41 items		–	x	–	x	x	[27]
Fear of falling (FES-I), 16 items			x	x	x	x	[28]
History of falls			during preceding year	during preceding 3 months	during preceding 3 months	during preceding 6 months	
Depressive symptoms (CES-D), 20 items			x	x	x	x	[36]
Pain	Presence of musculoskeletal pain		x	x	x	x	[38]
	Pain Interference Subscale from the Brief Pain Inventory (BPI)		x	x	x	x	[29]
Maximal isometric grip strength			x			x	
Perceived environmental barriers	PENBOM		x	x^a^	x	x	[31]
	Home environment		x	x	x	x	[47]
Perceived environmental facilitators	PENFOM		x	x^a^	x	x	[31]
	Exercise facilities		x	x^a^	x	x	
	Home environment		x	x	x	x	
Barriers to physical activity	Barriers (BOPA)		x	x^a^	x	x	[30, 48]
	Avoidance of moving outdoors		x	x^a^	x	x	[48]
Health, Cognition & demographics							
Health Status	Chronic diseases, current diagnosis (needing hospital care), treatment		^b^				
Medication			^b^	x	x	x	
Self-rated health			x	x	x	x	
Cognitive impairment	Cognitive status (CERAD)		x	–	–	–	[33, 34]
	Executive functions (TMT-A & -B)		x	–	x	–	[35]
Social contacts and socioeconomic status			x	–	–	–	
Weight, height			x	–	–	–	
Unintended weight loss			x	x	x	x	
Self-rated sensory functions	Vision, hearing		x	–	–	–	[49]
Standard care & rehabilitation			x	x	x	x	[38]
Use of social and health services			x	x	x	x	[38]

^a^Using Maptionnaire

^b^confirmed by a research nurse from medical records

### Outcome measures of physical activity and mobility

The primary outcome of this study is PA and sedentary time measured with a 3-dimensional accelerometer (Hookie AM20 Activity Meter, Hookie Technologies Ltd., Espoo, Finland and UKK RM42, UKK Institute, Tampere, Finland). The accelerometer is attached on the non-affected anterior thigh line with a transparent, adhesive film as typically used in wound healing (Opsite Flexigrid, Smith&Nephew, United Kingdom). Data are collected over six consecutive days. In the analyses, the crude accelerometer data are used for calculating the parameter of interest. Sedentary behavior (time spent still without moving, MAD <0.0167 g) is assessed from the raw acceleration data based on intensity. The intensity of PA is calculated as one-minute ranges of movement measuring an average of MAD of the resultant acceleration and converted into metabolic equivalents (MET). Using METS, PA is classified into light, moderate and vigorous PA. MAD-based cut-points for light, moderate and vigorous locomotion against VO_2_ across a range of walking speeds are validated in our treadmill tests. In addition to accelerometer measurements, the participants keep PA diary on data collection days.

Level of PA over the previous month is assessed with the self-report scale by Grimby [22], which has been translated into Finnish and further modified and validated [23, 24]. The 7-point scale describes the level of PA as follows: 0) mostly resting, or lying down, 1) hardly any activity, mostly sitting, 2) light physical activity, such as light household tasks, 3) moderate physical activity for about 3 h a week: walking longer distances, cycling and domestic work, 4) moderate physical activity for at least 4 h a week or heavier physical activity 1–2 h a week, 5) heavier physical activity or moderate exercise for at least 3 h a week, and 6) competitive sports. Self-reported PA is assessed at 12 months in addition to the baseline, 3- and 6- month measurement points.

Mobility is measured using the Short Physical Performance Battery (SPPB). The SPPB evaluates balance, mobility and muscle strength by examining an individual’s ability to stand in different positions, time taken to walk 4 m, and time taken to rise from and sit down on a chair 5 times. Each test is scored between 0 and 4, yielding a maximum score of 12. The SPPB is a validated and frequently used tool in older people, with low SPPB sum scores predicting falls, loss of independence and mortality [25].

Perceived difficulties in mobility (in walking outdoors, in walking 2 km and 500 m, climbing up 1 flight of stairs) are assessed using a structured questionnaire with the following response categories: 1) able without difficulty, 2) able with some difficulty, 3) able with a great deal of difficulty, 4) unable without the help of another person, and 5) unable to manage even with help. We assess self-rated mobility by asking “How would you describe your mobility?” using a 4-point scale (very good, good, poor or very poor). The use of an assistive device for mobility is rated for seven listed assistive devices with the response options: 1) no, 2) yes, only indoors, 3) yes, only outdoors, and 4) yes, both indoors and outdoors.

### Secondary outcomes

Life space mobility is measured using the University of Alabama at Birmingham Study of Aging Life Space Assessment, LSA [26]. The LSA comprises 15 items and assesses mobility through the different life-space levels (distance), which the participant reports having moved through during the four weeks preceding the assessment. For each life-space level (bedroom, other rooms, outside home, neighborhood, town, beyond town), participants are asked how many days a week they attained the level and whether they needed help from another person or from assistive devices. Scores range from 0 to 120 with higher scores indicating a larger life-space. Life-space one-month prior to hospital admission is assessed retrospectively during hospitalization. We also ask participants about their life-space mobility during the two weeks immediately following discharge from hospital. We want to evaluate life space shortly after returning home but we do not want to burden our participants with an additional questionnaire or home visit at one month after their hospital discharge. During the measurements at 3 months, after the 6-month intervention, and at the 12-month follow-up, the original 4-week period preceding the assessment is used in the life-space assessment questionnaire.

The Impact on Participation and Autonomy (IPA) questionnaire [27] is a validated questionnaire designed to assess perceived autonomy and participation in various clinical populations, including older people. It consists of five domains: social relations, autonomy in self-care, autonomy outdoors, family role, and work and educational opportunities. The response categories range from 0 (very good) to 4 (very poor). A sum score is calculated, with a higher score indicating more restrictions in participation.

Fear of falling is assessed by Fall Efficacy Scale International (FES-I) [28]. Sixteen questions on concerns about the falling when performing different activities are scored on a 4-point scale ranging from “not at all concerned” to “very concerned”. The scores are added up to calculate a total score that ranges from 16 to 64. A higher score indicates a greater fear of falling. Information on falls (“Have you fallen or slipped during the previous year/previous three months?” yes/no) and injurious falls (“If yes, how many times did you need to be treated by a doctor?”) is also collected.

Disturbing musculoskeletal pain in the low back, hip, knee, ankle and foot is assessed by a questionnaire. Musculoskeletal pain is assessed with the question "Have you suffered from pain in the low back, hip, knee, ankle or foot region daily during the preceding month? Has the pain compromised your mobility?" The three alternative response options are: 1) no; 2) yes, but the pain does not limit my mobility; and 3) yes, the pain limits my mobility. We assess also assess the impact of pain on daily function using the Pain Interference Subscale from the Brief Pain Inventory (BPI) questionnaire [29]. Participants are asked to rate on a scale from 0 to 10 the extent to which pain has interfered with their general activity, mood, walking ability, normal work, relations with other people, sleep and enjoyment of life during the past 24 h. Grip strength of the dominant hand is measured at baseline and at the 6-month follow-up using a Jamar digital hand dynamometer in the sitting position with the elbow in 90 ° flexion close to the body.

### Barriers and facilitators to physical activity

The questionnaire on barriers to PA comprises 17 items under the themes of poor health, fear and negative experiences, lack of knowledge, lack of time and interest, lack of company and unsuitable environment [30]. Each item is rated as yes or no. Barriers and facilitators to mobility in the outdoor (PENBOM and PENFOM) and entrance environments of the home are examined as perceived by the participants, using standardized questionnaires developed in earlier studies [31, 32]. Three months after hospital discharge, environmental features hindering and facilitating outdoor mobility reported by participants will be located on a map using cloud-based Maptionnaire software (Mapita LTD, Helsinki, Finland).

### Review of medical data and health status and demographics

Data on age, gender, marital status, living conditions and education are collected. Self-reported height and weight are recorded and a question about unintentional weight loss of five kg or more is included in the questionnaire. When possible, weight is measured using a standard procedure at the health center hospital. Body mass index is calculated as weight (kg)/height × meters^2^. Perceived sensory functions (vision and hearing) are assessed using a structured questionnaire. At baseline, cognitive status is assessed by the Consortium to Establish a Registry for Alzheimer’s Disease (CERAD) neuropsychological battery [33], which includes a Mini Mental State Examination (MMSE) [34], Executive functions are assessed by Trail Making Tests A and B [35] at baseline and six-month follow-up. Depressive symptoms are measured by the Center for Epidemiological Studies-Depression Scale (CES-D) [36] at baseline and at 3, 6 and 12 months after baseline. Self-rated health is evaluated by the question “How would you describe your health?” using a 4-point scale (very good, good, poor or very poor).

Information on use of formal and informal care and form of dwelling is collected by a questionnaire. At the end of the study, we will the collect information on the stay in hospital and use of formal care from the registers of the health center hospital and national register on the use of health and social services.

### Standard care

At three months after hospital discharge, all participants are interviewed with structured questions on advice, recommendations and possible planned programs concerning the rehabilitation they received post discharge. Typically, older people receive written information on home exercises and safe walking and instructions on how to rise from a chair from a physiotherapist in the hospital ward before discharge to home. Half of the participants will have received standard care alone (control group).

### ProPA intervention

The ProPA intervention study includes a multi-component home rehabilitation intervention program and standard care. The ProPA intervention is an individually tailored 6-month PA and rehabilitation program aimed at promoting physical activity and restoring mobility after hospital discharge. A detailed description of the rehabilitation program is presented in Table 2. The idea for the program arose from the OTAGO Exercise Program designed to prevent falls in older people [37] and ProMo, a previous randomized controlled trial [38] that was successful in restoring mobility [18] and physical activity [19] among community-dwelling older people with a recent hip fracture.

**Table 2:** Flow and content of Propa intervention

	Aim	Intervention content	Method
1. Home visit (week 1)	Introduction to the rehabilitation program, duration, GAS method and aims of the study	Current health status, chronic diseases, falls, living environment and use of a walking aid are evaluated. Goal setting is initiated	Interview, information on paper form is given on helping aids, hip pants, shoes and ancillary equipment
2. Home visit (week 2)	Muscle strength program	Individual exercise program to be implemented according to the OTAGO protocol (strength, balance, functional training)	Exercise training, counseling
3. Home visit (week 3)	Functional exercise program (e.g. walking, climbing stairs). Goals of rehabilitation are assessed to fit the participant’s current situation	Individual exercise program to enhance functional capacity and independent ADL functions. Advice for non-medical solutions to increase pain-management skills	Exercise training, interview, counseling
4. Home visit (week 4)	Balance program with progressive balance movements on an individual level (with help of ancillary equipment if needed)	Individual exercise program to increase and maintain balance and agility	Exercise training, goal updating, counseling
1. Call (week 6)	Encouragement to pursue goals and information given if needed	Evaluation of current situation, progress with the training program and their health status	Counseling goal updating
5. Home visit (week 8)	Functional training and progression on each program. Light resistance band training.	Training program is evaluated to fit the participant’s current situation.	Exercise training, interview, goal setting
2. Call (week 10)	Encouragement to pursue goals and information given if needed	Participants are asked about their current situation, progress with the training program and their health status	Counseling
6. Home visit (week 12)	Possibility to train outside of the participants home, e.g., communal gym, swimming pool, elderly exercise group or home-based medium resistance band training	Physical activity counselling, goal setting	Exercise training, counseling, motivational interview to discover new training possibilities
3. Call (week 16)	Encouragement to pursue goals and information given if needed	Evaluation of current situation, progress with the training program and their health status	Counseling
7. Home visit (week 20)	Evaluation of rehabilitation period and plan for future	The success of the goals set beforehand are appraised and new goals set for the future. Evaluation of the physical activity plan. Motivation to continue physically active lifestyle	Counseling

Within two weeks from randomization, a physical therapist initiates the intervention in the participant’s home. The rehabilitation program includes seven home visits, a home exercise program according to the OTAGO protocol, and three phone calls by a physiotherapist. The home visits are targeted for weeks 1, 2, 3, 4, 8 12 and 20, week1 being the start of the rehabilitation program. Booster phone calls to reinforce adherence to the home exercise regimen and physical activity recommendations are targeted for weeks 6, 10 and 16. The participant goes through the OTAGO home exercise program together with the physiotherapist and, in addition, receives instructions in writing. The program, with accompanying illustrations, has been described in more detail earlier [39]. The program is to be performed three times a week. It includes strengthening exercises for the lower limb muscles, balance training, and walking exercises and will be upgraded 4 to 5 times. Progression of the strengthening exercises is implemented with resistance bands. All participants keep an exercise diary on the exercise program during the intervention.

#### Physical activity counseling

Goal Attainment Scaling (GAS) [40] is applied to set one or more PA-related goals. During the first visit, the method is presented to the participants. Their own goals and wishes are discussed to enable them to set goals based on the SMART principle (specific, measurable, achievable, realistic and time-based) [41].

Individual face-to-face PA counseling with a personalized PA plan takes place after three months in the participants’ homes. Motivational interviewing techniques will have been used during the counseling sessions to help participants to find inner motivation for adopting an active lifestyle, overcome barriers and detect sedentary behavior patterns. The topics covered during the session include the participant’s earlier and present PA level, the participant’s interest in returning to her or his previous activities, the possibility to starting a new type of PA or exercise, and guidance on how to be active in performing everyday chores. The problem-solving method is used to address perceived obstacles to PA. Participants are also given information on the PA courses and facilities offered by the municipality. To practice the exercise program, they are also given an opportunity to visit a gym or swimming pool with the physiotherapist.

### Statistical analysis

All analyses will be performed using the intention-to-treat principle. Means, standard deviations and frequencies will be calculated for the demographic variables. Normality of the distributions will be tested. At baseline, the significance of differences between the intervention and control group will be tested by cross-tabulation and chi-square tests in the case of discrete variables, by Student’s t-test for independent samples for normally distributed data and by Mann-Whitney U-test for non-normally distributed continuous data. Associations between variables will be analyzed using Pearson’s correlation coefficient. The effects of the intervention will be assessed using repeated measures ANOVA, covariance analysis and linear mixed models. Generalized estimating equations (GEE) models will be used to analyze differences in changes in prevalence over three time points. The level of statistical significance will be set at *p* < 0.05 (two-sided). In all future publications, data will be reported following the criteria recommended by the CONSORT guidelines [42].

## Discussion

The purpose of this paper was to describe the study protocol of the ongoing randomized controlled trial. The aim of the project is to examine whether an individual home-based rehabilitation program has positive effects on PA and recovery of mobility in older community-dwelling people after acute hospitalization for a musculoskeletal injury or disorder.

Reduction in sedentary time and engaging in daily PA in the post-hospital period is critical for recovery [43], and it may also help prevent future hospitalization. The overarching health care challenge is to encourage older people who have recently been discharged from hospital to return to their usual activities of daily living and engage in regular PA. This project is thus a call for action in designing appropriate solutions to encourage older people to be as physically active as their functional status allows.

The study design is based upon the results of our earlier pilot study and issues reported from previously published study in which the effectiveness of home-based rehabilitation program on mobility was shown among hip fracture patients [15, 44]. Here, we extend the previous intervention in further trial with a heterogeneous high-risk population. This protocol differs from that of the previous study in three key areas: in patient selection, intervention design and outcome measurements. Our preliminary data included an observational study with 55 older people aimed at testing our preliminary outcome measures and improving our understanding of the barriers and enablers to taking part in a rehabilitation program after an acute hospital stay. Our research team includes scientists with long experience in aging studies, and clinicians who work daily with the geriatric population.

The intervention is designed to increase PA, which might enhance patients’ personal life-space mobility and physical functioning. For this reason, the primary outcomes are PA and sedentary behavior measured by a 3D accelerometer and mobility measured using the SPPB. However, to gain detailed information on older persons’ life situations and participation in society after an acute medical challenge, other outcome measures are also important. To our knowledge, little is known on the effect of easily adoptable home-based rehabilitation programs on the PA of participants recently discharged from hospital after a musculoskeletal injury or surgery. In this time period specific patient groups have just initiated the recovery process, but are still weak and in need of assistance to complete their recovery. To contribute to meeting this need, we propose a home-based exercise program that may overcome some of the barriers to participation in rehabilitation, such as difficulties in using public transportation and lack of support for going outside the home without help. The intervention is supported by a physiotherapist regularly, as we consider that the home-visits during the follow-up period, will support patients to achieve their goals and succeed with the rehabilitation program designed together with them. Specially patients with cognitive problems or who have lack of motivation. This program might have potential to be integrated to the public health system and add value to the hospitals discharge protocol for the benefit of older people.

We are aware that it is challenging to run an intervention for older people who also suffer from multiple diseases and whose range of functional capacity is wide. Nevertheless, it is important to conduct a real-life study and develop an intervention that could also help promote daily PA and minimize mobility decline in older adults with low functional capacity. This project has the ability to bring out new practices regarding PA programs for older people after acute hospitalization due to musculoskeletal problems. We believe that the knowledge gained from this study will be of value to the scientific community and help in the development of rehabilitation strategies in hospitals, primary health care and the home environment among older people.

## Abbreviations

3D: three-dimensional
BPI: the Brief Pain Inventory questionnaire
CERAD: Consortium to Establish a Registry for Alzheimer’s Disease
CES-D: the Center for Epidemiological Studies-Depression scale
CONSORT: Consolidated Standards of Reporting Trials
FES-I: Fall Efficacy Scale International
GAS: Goal Attainment Scaling
GEE: Generalized estimating equations
IPA: The Impact on Participation and Autonomy questionnaire
LSA: the University of Alabama at Birmingham Study of Aging Life Space Assessment
MAD: Mean amplitude deviation
MET: Metabolic equivalent
MMSE: Mini Mental State Examination
OTAGO: Otago Exercise Programme to prevent falls in older adults
PA: Physical activity
ProMo: Promoting mobility after hip fracture
ProPA: Promotion of physical activity and mobility among older people with musculoskeletal disorders rehabilitation program
RCT: Randomized controlled trial
SPPB: the Short Physical Performance Battery
VO2: Oxygen consumption
